# Pacing and Sensing of Human Heart for over 31 Years with the Same Apparatus (Generator and Lead)

**DOI:** 10.1155/2015/796954

**Published:** 2015-10-26

**Authors:** Evangelos Papasteriadis, Panagiotis Margos

**Affiliations:** 1st Cardiology Department, General Hospital of Nikea, 18454 Piraeus, Greece

## Abstract

Several patients receive a permanent pacemaker in a relatively young age, with multiple subsequent reoperations for pacemaker replacement. Pulse generator replacement is an invasive procedure, associated with the risk of various complications, mainly infection and skin erosion. A case of an extremely long-lasting pacemaker with a totally uneventful longevity period over 31 years is presented. The explanation for this quite rare pacemaker longevity (possibly unique) is analyzed and discussed.

## 1. Case Presentation

A 45-year-old woman was referred to our hospital from a provincial hospital, due to sick sinus syndrome (tachy-brady syndrome causing fainting attacks). Clinical examination and laboratory tests were normal. A VVI (multiprogrammable) Siemens Elema 668 (SN 336801220) pulse generator with a passive fixation carbon tip unipolar lead 412S/60 of the same company was implanted in her on October 10, 1983. This was a nominal high voltage (5 V) device, in contrast with the lower full-charge voltage of the new modern generators (about 2.76 V). The implantation procedure (lead tip placed at the right ventricular apex) was uncomplicated, with the following acceptable parameters: R wave (sensing) 8 mV, pacing threshold 0.7 V/0.5 ms, and pacing resistance 750 Ω. This is a passive fixation, 60 cm long unipolar lead with a silicone rubber as an insulation and activated vitreous carbon, porous tip. This type of electrode provides, among other advantages, lower current and voltage for stimulation acutely and chronically [1]. The generator was programmed with the nominal program “on” (output 5 V/0.5 ms pacing at 70 bpm, Vario off).

**Figure 1:**
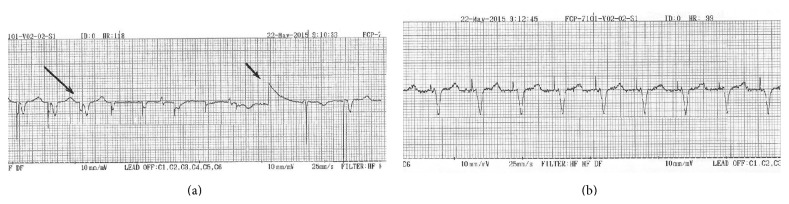
(a) “Vario” method for pacing threshold evaluation with automatically decreased consecutive steps of pacing voltage output. Spike deflection (small arrow) presents zero voltage. Backwards from that zero point, each spike presents increased voltage with steps of 0.15 V. The 5 spikes at the left of zero point with no capture represent steps of 0.15–0.75 V. Capture is succeeded by the 6th spike before zero point (large arrow), with output 0.9 V/0.5 ms (pacing threshold on May 22, 2015). (b) Magnet rate of 99 bpm on May 22, 2015 (recommended replacing magnet rate ≤ 85 bpm).

The patient was discharged 24 hours later and she had follow-up visits every six months. Fifteen months after implantation, on January 1985, Vario was assigned to “on” mode. In the Vario mode, which was historically the first manually applied algorithm for pacing threshold measurement, magnet application results in 16 asynchronous beats at the magnet rate of 100 bpm, followed by 16 asynchronous beats at a rate of 125 bpm. During the 16 beats at 125 bpm the voltage output is reduced by 1/15 progressively until zero output is reached [1]. In addition to Vario “on” mode, output was decreased 2.5 V/0.5 ms as there was stable pacing threshold < 1 V/0.5 ms (safety margin > 2/1). This threshold remained stable for more than three decades (Figure 1(a)). The patient's European Pacemaker Registration Card is completed with the follow-up data, during this period of time.

**Figure 2:**
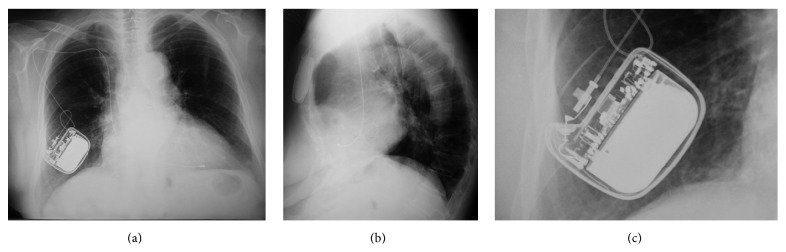
((a), (b)) Chest X-ray (May 22, 2015) of anteroposterior and lateral views. (c) Enlargement of the pulse generator figure, showing the initial generator-lead connection, without the presence of IS-1 lead connector.

Her last visit in our outpatients pacemaker clinic was on May 22, 2015, 31 years since implantation. ECG revealed pacing rhythm with underlying atrial fibrillation. The application of a magnet showed ventricular capture in a magnet rate of 99 bpm (Figure 1(b)). Pacing threshold was estimated with the use of Vario system at 0.9 V/0.5 ms, remarkably close to the initial threshold during the implantation. The chest X-ray showed normal findings, with no evidence of lead fracture (Figure 2). Next follow-up visit is scheduled for November 2015.

## 2. Discussion

The main problem of pacemaker replacement is the risk of infection and increases twofold in replacements [2], mainly due to impaired blood flow and subsequent immunodeficiency in the fibrotic environment of an old pacemaker pocket. Iatrogenic (operator-related) lead failure due to inappropriate manipulation may also occur during the procedure. As a consequence, serious problems may emerge during the procedure of pacemaker replacement [2–4].

Currently, the longevity of a pulse generator ranges between 5 and 14 years, with a mean duration of 7-8 years [5]. The systematic use of lithium-iodine battery (the main power source for the pacemakers which replaced mercury battery) nowadays runs its fifth decade, with no significant technological progress in this field lately, despite the increasing number of pacemaker implantations worldwide [6]. The problem of multiple reoperations is intensified due to the increased implantation rate of more energy-consuming devices with significantly shorter battery longevity, like biventricular pacing systems or implantable cardioverter-defibrillators. Despite frequent follow-up and appropriate programming of the device with the use of sophisticated, energy-saving algorithms [7], the goal of longer-lasting pacemaker generators still remains an issue.

Review of the literature provides poor data, regarding cases of very long-lasting pacemakers. A relatively recent report of a pacemaker with a duration of 26.3 years claims the title of the longest-lasting pacemaker worldwide [8]. In the same publication, the authors report a specific generator model with a quite long average duration (of 19.2 years).

Basic physics provide data regarding energy consumption of an electrical circuit: energy (*E*, in Joules) = *V* ^2^ × *t*/*R* (*V* = voltage, *t* = time, and *R* = resistance). Regarding our patient, parameters “*t*” and “*R*” remained stable as we had “Vario on” mode (January 1985), with no significant changes during the long period of 31 years. Reducing by 50% the voltage in a generator is the key point of low energy consumption and subsequent extreme generator longevity. Theoretically, according to the above equation, a 5 V generator provides 4-fold energy in comparison to a 2.5 V generator! (5^2^/2.5^2^ = 4). In agreement with this scenario, it has been reported that battery capacity (which is analogous to voltage) is the strongest determinant of increased pacemaker longevity [7].

Low programmed output, low percentage of long-term pacing, and high-impedance leads are also major determinants of generator longevity [5, 7, 9, 10]. Unfortunately, we have no data for V-pacing percentage in our patient, provided the obsolete software of the device (output was programmed at 2.5 V/0.5 ms, as mentioned previously). It is well known that interrogation of many modern devices frequently reveals quite low percentage of pacing in patients with sick sinus syndrome. Sophisticated algorithms that minimize ventricular pacing of DDDR pacemaker contribute to this phenomenon. Autocapture is also a modern application that allows safe pacemaker function in a quite low and energy-saving output, occasionally lower than 1 V/0.4 ms. Additionally, many patients with permanent atrial fibrillation and VVIR pacemakers present steadily low percentage of V-pacing. Nevertheless, longevity of modern pacemakers is rarely over 12 years, even in patients with very low percentage of pacing and autocapture mode [5, 7, 11]. The transition from high voltage to lower voltage pacemakers was obviously decision of the manufacturers, probably due to technical issues. In the present case, the reduced output (2.5 V/0.5 ms) of the high voltage (5 V) old device indicated energy saving in a device with high battery capacity, providing the main explanation for the extreme longevity beyond 31.6 years.

In conclusion, reducing the pulse generator's output and keeping constant pulse width, battery's life can be extended, provided that the safety margin is greater than 2 : 1 and the patient has a regular follow-up. Pacing with 2.5 V/0.5 ms is feasible for a long period of time, even for more than 3 decades. Application of modern energy-saving algorithms also contributes to this goal.
